# Prognostic Value of the Geriatric Nutritional Risk Index in Non-Small Cell Lung Cancer Patients: A Systematic Review and Meta-Analysis

**DOI:** 10.3389/fonc.2021.794862

**Published:** 2022-01-18

**Authors:** Haoyu Wang, Cui Li, Ruiyuan Yang, Jing Jin, Dan Liu, Weimin Li

**Affiliations:** ^1^ Department of Respiratory and Critical Care Medicine, West China Hospital, Sichuan University, Chengdu, China; ^2^ Institute of Respiratory Health, Frontiers Science Center for Disease-Related Molecular Network, West China Hospital, Sichuan University, Chengdu, China

**Keywords:** lung cancer, serum albumin, body weight, Geriatric Nutritional Risk Index, survival, prognostic value

## Abstract

**Background:**

Novel evidence showed that the Geriatric Nutritional Risk Index (GNRI) may lead to poor prognosis of human cancers. Therefore, we conducted a meta-analysis to explore the impact of GNRI in lung cancer and its prognostic value.

**Methods:**

We searched the databases of PubMed, Web of Science, Embase, Scopus, and Cochrane Library up to July 2021 for relevant research and merged the hazard ratios (HRs) and 95% confidence intervals (CIs) to evaluate the association between GNRI and overall survival (OS), cancer-specific survival (CSS), and recurrence-free survival (RFS) in patients with lung cancer.

**Results:**

Eight studies involving 2,399 patients were included in our primary meta-analysis. The results indicated that lower level of GNRI was associated with poorer OS, RFS, and CSS of lung cancer patients (OS: HR = 1.99, 95% CI: 1.68–2.35, p < 0.0001; RFS: HR = 2.34, 95% CI: 1.11–4.95, p = 0.0258; CSS: HR = 2.45, 95% CI: 1.43–4.18, p = 0.0011). The association was robust after subgroup analysis and sensitivity analysis.

**Conclusions:**

GNRI may be a prognostic factor of lung cancer, which can lead to poorer survival. However, more prospective studies are necessary to confirm the results.

**Systematic Review Registration:**

International Prospective Register of Systematic Reviews (PROSPERO), identifier CRD42021269574.

## Introduction

The novel cancer statistics revealed that 2.2 million new cases and 1.8 million new deaths of lung cancer occurred, which demonstrated that lung cancer is still the leading cause of cancer death (1, 2). Though breast cancer has now been the form of cancer with the highest incidence, the disease burden of lung cancer still cannot be ignored, with a bad prognosis of extremely low 5-year relative survival rate less than 20% (2). Although the diagnostic and therapeutic strategies for lung cancer are burgeoning, there are very few biomarkers evaluating the prognosis of lung cancer patients (3). Therefore, an efficient and convenient method to predict the prognosis of lung cancer patients is indispensable. Previous studies demonstrated that malnutrition is associated with the cachexia of cancer patients (4) and can even affect the complications and survival of human malignancies (5), and several nutritional biomarkers were applied to predict the survival of lung cancer patients, such as the Prognostic Nutritional Index (PNI) (6) and Controlling Nutritional Status (CONUT) (7, 8). The Geriatric Nutritional Risk Index (GNRI) (9), a novel index designed for assessing the nutritional status of elderly patients, has also shown good prognostic value in tumor patients (10, 11). However, whether pretreatment GNRI is a good prognostic factor for lung cancer has not been systematically evaluated; thus, in this study, we explored the impact of GNRI on lung cancer prognosis by a meta-analysis.

## Methods

### Protocol and Registration

The current meta-analysis was conducted according to the Preferred Reporting Items for Systematic Reviews and Meta-Analyses (PRISMA) statement (12) and was registered at the International Prospective Register of Systematic Reviews (PROSPERO): number CRD42021269574.

### Search Strategy

The systematic search for the eligible literature was performed in the following databases including PubMed, Web of Science, Scopus, Embase, and the Cochrane Library up to July 2021. The following key words were used to retrieve potential research: “pulmonary neoplasms,” “lung cancer,” “geriatric nutritional risk index,” “GNRI,” “survival,” and “prognosis.” Additional articles were manually retrieved from the reference lists of relevant research, and the included articles were restricted to English. The detailed search strategy for PubMed was presented in **Supplementary Material 2**.

### Eligibility Criteria

Studies were included if they met the following criteria: 1) All patients were pathologically diagnosed with non-small cell lung cancer; 2) Studies investigated the prognostic value of GNRI that was calculated by pretreatment albumin level, pretreatment weight, and ideal weight; 3) The outcomes included the OS with hazard ratios (HRs) and corresponding 95% confidence intervals (95% CIs); 4) retrospective or prospective studies with the full-text paper published before July 2021.

Studies were excluded if they met the following criteria: 1) reviews, conference abstracts, case reports, letters, or comments; 2) laboratory research of clinical samples, cell lines, or animals; 3) insufficient data of GNRI or lack of control; 4) Full-text paper written in English was not available.

### Data Extraction

Two researchers extracted the following data from the eligible studies independently: family name of the first author, year of publication, study design, region, median follow-up (in months), sample size, GNRI cutoff value, tumor stage, therapy, and outcomes with HRs and their corresponding 95% CIs. Any disagreement was resolved by discussion and consensus.

### Risk of Bias Assessment

The risk of bias assessment was conducted by the Newcastle–Ottawa quality assessment Scale (NOS), and studies labeled with 6 points or higher were regarded as high-quality studies.

### Statistical Analysis

Statistical analysis was performed by R (version 4.0.3) and R Studio (version 1.3.1). HRs from the multivariate analysis were used wherever available, and HRs from univariate analysis were substituting only if the results of univariate analysis were provided. Besides, HRs of multiple groups were merged using a fixed-effects model for the main analysis if HRs of different GNRI levels were provided independently. In addition, HRs were estimated by applying the method of Tierney in case they were not provided directly. Pooled HRs and 95% CIs were combined with the random effects or fixed-effects model according to heterogeneity. Heterogeneity was assessed by forest plots, Q tests, and I^2^ statistics. Significant heterogeneity was defined as p-value <0.05 and I^2^ > 50%, and the random-effects model was used under this condition. Otherwise, we chose the fixed-effects model. Subgroup analyses were performed to investigate potential confounding factors of this meta-analysis. Sensitivity analysis was conducted by excluding each study independently from our meta-analysis to find out the overrepresentation of every single study. Publication bias was evaluated by Begg’s test (13), Egger’s test (14), and funnel plots. A trim-and-fill method was used to modify our results of meta-analysis if significant publication bias existed (15). p-value <0.05 was considered to be statistically significant.

## Results

### Literature Search and Risk of Bias Assessment

**Figure 1:**
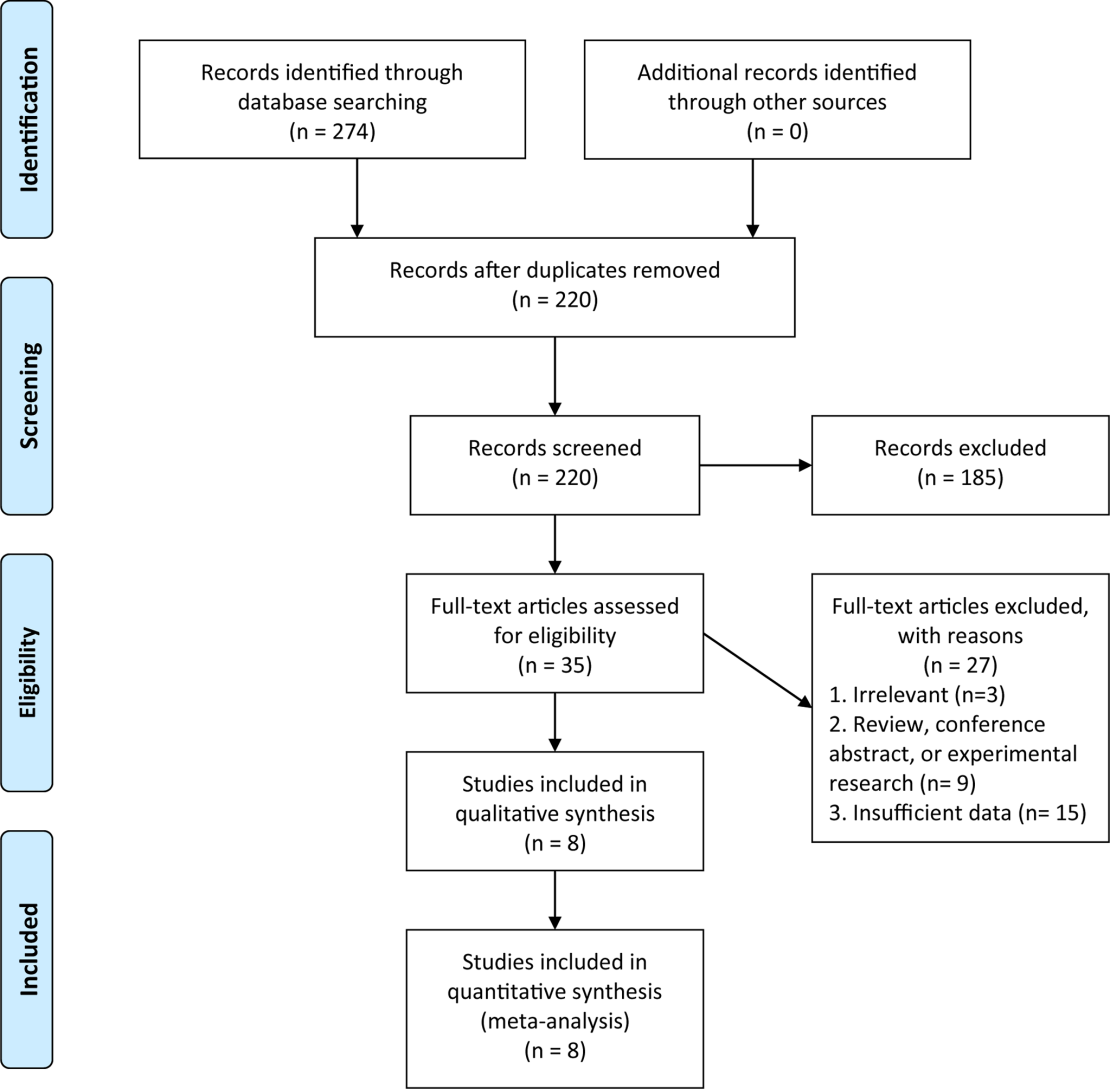
Preferred Reporting Items for Systematic Reviews and Meta-Analyses (PRISMA) flow diagram of the literature search in this meta-analysis.

According to our search strategy, a total of 274 potential publications were obtained, and 220 separate articles were further screened after removing duplicates. According to titles and abstracts, 35 studies were selected for the screening of full-text version, and finally, 8 studies were included in our present meta-analysis. The NOS scores of these studies ranged from 6 to 9, which represented a low risk of bias. The PRISMA flow diagram and checklist of this meta-analysis were presented in **Figure 1** and **Supplementary Material 1**, respectively.

### Characteristics of the Included Studies

The main characteristics of the included studies were displayed in **Table 1**. A total of 8 articles including 2,399 patients were enrolled in our meta-analysis (16–23); 1 study was prospective (18), while the others were retrospective. Besides, 6 studies were conducted in Japan (16, 17, 20–23), whereas 2 studies were in China (18, 19); 5 studies enrolled patients with Stage I–III lung cancer, while the remaining studies enrolled patients with Stage III–IV lung cancer. The sample size of these studies varied from 85 to 739, and the main cutoff value of GNRI was 98. All studies defined OS as the time from inclusion to the date of death or last follow-up.

**Table 1:** Main characteristics of the studies included.

Source	Design	Region	MFP (months)	Sample size	Stage	Therapy	Cut-off value	Outcome	NOS
Shoji et al. (16)	RO	Japan	58	141	I	Surgery	98	OS, CSS, RFS	8
Hino et al. (17)	RO	Japan	40.63	739	I-III	Surgery	98	OS, CSS	7
Peng et al. (18)	PO	China	28	257	III-IV	Mixed	98	OS	7
Tang et al. (19)	RO	China	17.2	144	IV	Mixed	97	OS	6
Asakawa et al. (20)	RO	Japan	>60	286	I-IIA	Surgery	102.1	OS	8
Sonehara et al. (21)	RO	Japan	NA	85	IV	ICI	89.5	OS, PFS	6
Shoji1 et al. (22)	RO	Japan	51	272	I-III	Surgery	98	OS	9
Takahashi et al. (23)	RO	Japan	46	475	I-III	Surgery	101	OS, RFS	7

MFP, median follow-up; NOS, Newcastle-Ottawa quality assessment Scale; RO, retrospective study; PO, prospective study; ICI, immune checkpoint inhibitor; OS, overall survival; CSS, cancer-specific survival; RFS, recurrence-free survival; PFS, progression-free survival.

### Association Between Geriatric Nutritional Risk Index and Survival in Lung Cancer Patients

**Figure 2:**
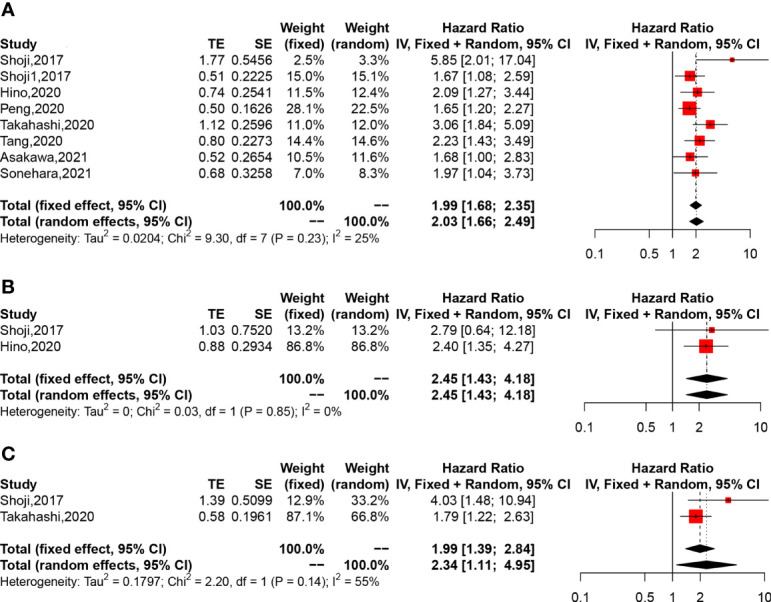
The forest plot of the association between Geriatric Nutritional Risk Index (GNRI) and the clinical outcome of non-small cell lung cancer patients. **(A)** Forest plot of overall survival (OS). **(B)** Forest plot of cancer-specific survival (CSS). **(C)** Forest plot of recurrence-free survival (RFS).

A total of 2,399 patients based on 8 studies contributed to our primary analysis. The results of the pooled analysis revealed that a lower pretreatment GNRI was associated with poorer OS in lung cancer patients with low heterogeneity (HR = 1.99, 95% CI: 1.68–2.35, p < 0.0001, I^2^ = 25%, p = 0.23) (**Figure 2A**). Besides, a lower pretreatment GNRI was also correlated to poorer cancer-specific survival (CSS) (HR = 2.45, 95% CI: 1.43–4.18, p = 0.0011, I^2^ = 0%, p = 0.85) (**Figure 2B**) and recurrence-free survival (RFS) (HR = 2.34, 95% CI: 1.11–4.95, p = 0.0258, I^2^ = 55%, p = 0.14) (**Figure 2C**) with very few studies included.

### Subgroup Analysis

To detect the potential origin of the heterogeneity among the included studies, we subsequently performed subgroup analyses stratified by region, tumor stage, therapy, sample size, GNRI cutoff value, median follow-up (months), and publishing time. As shown in **Table 2**, the relationship between GNRI and OS in lung cancer patients showed a similar trend in most subgroups. However, only studies published in 2017 showed a significantly high heterogeneity (I^2^ = 78%, p = 0.03).

**Table 2:** Results of subgroup analyses.

	N	Association		Heterogeneity	
		HR (95%CI)	p	I^2^	p
**Region**					
Japan	6	2.12 (1.69-2.64)	<0.01	33%	0.19
China	2	1.83 (1.41-2.37)	<0.01	15%	0.28
**Tumor stage**					
I-III	5	2.14 (1.69-2.71)	<0.01	46%	0.12
III-IV	3	1.85 (1.45-2.35)	<0.01	0%	0.54
**Therapy**					
Surgery	5	2.14 (1.69-2.71)	<0.01	46%	0.12
Non-surgery	3	1.85 (1.45-2.35)	<0.01	0%	0.54
**Sample size**					
≤200	3	2.38 (1.69-3.37)	<0.01	36%	0.21
>200	5	1.88 (1.59-2.28)	<0.01	17%	0.31
**Cut-off value**					
≤97	2	2.14 (1.49-3.09)	<0.01	0%	0.75
>97	6	1.95 (1.61-2.36)	<0.01	44%	0.11
**Median follow-up (months)**					
≤36	2	1.83 (1.41-2.37)	<0.01	15%	0.28
>36	5	2.14 (1.69-2.71)	<0.01	46%	0.12
NA	1	1.97 (1.04-3.73)	0.04	/	/
**Publishing time**					
2017	2	2.00 (1.33-2.99)	<0.01	78%	0.03
2020	4	2.04 (1.66-2.52)	<0.01	30%	0.23
2021	2	1.79 (1.20-2.68)	<0.01	0%	0.70
**Overall**	**8**	**1.99 (1.68-2.35)**	**<0.01**	**25%**	**0.23**

NA, not available. The bold values are pooled results of the hazard ratio, 95% confidence interval, I2 value, and p values for the primary analysis, which were also presented in **Figure 2A**.

### Sensitivity Analysis

**Figure 3:**
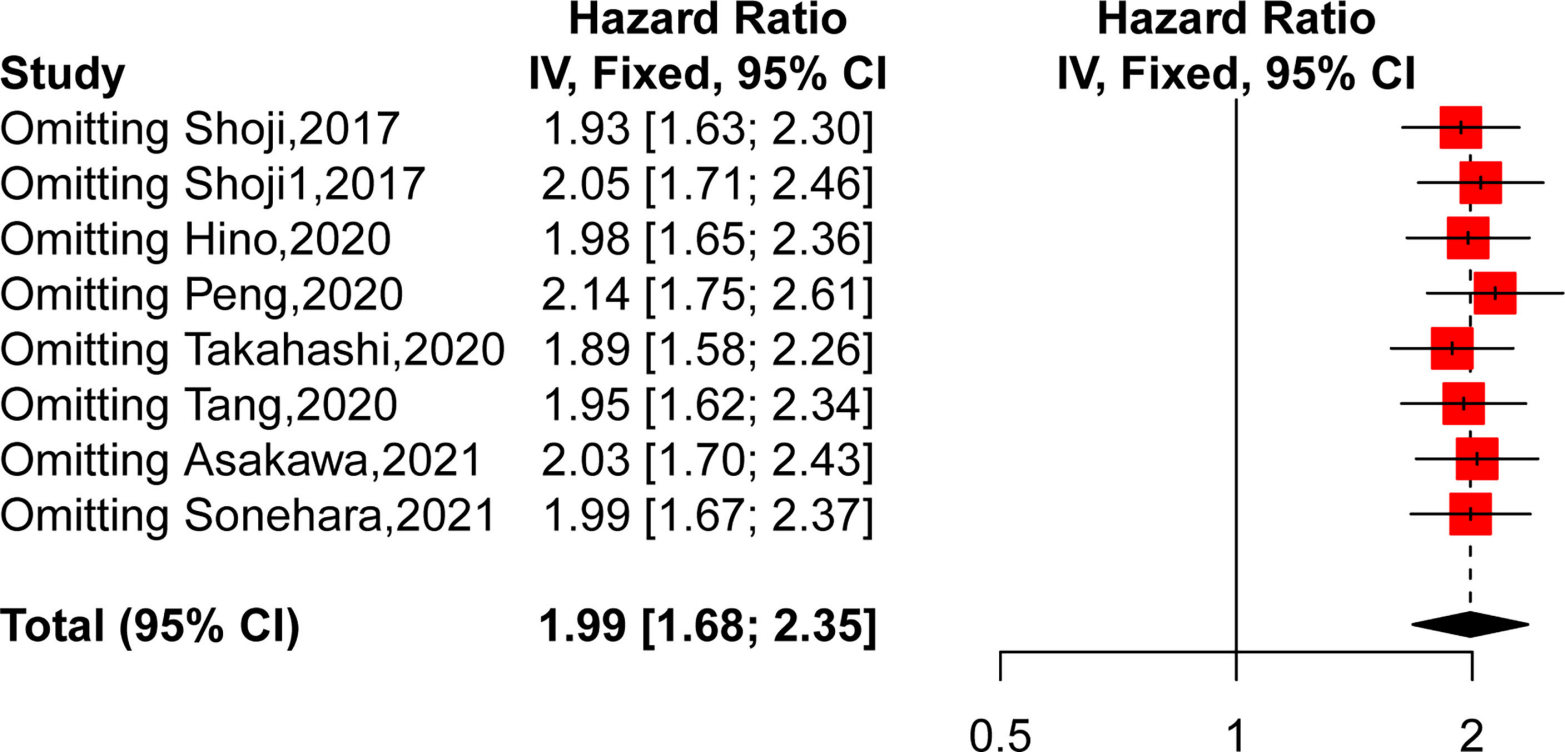
Sensitivity analysis by excluding each study from the meta-analysis.

We then performed a sensitivity analysis to further explore the source of heterogeneity by removing each study from the meta-analysis independently. As shown in **Figure 3**, the pooled HRs demonstrated that our results were robust. However, after removing the study of Shoji, the heterogeneity decreased obviously (HR = 1.9346, 95% CI: 1.63–2.30, p < 0.0001, I^2^ = 0%, p = 0.51), which suggested that this publication might be the main source of heterogeneity.

### Publication Bias

**Figure 4:**
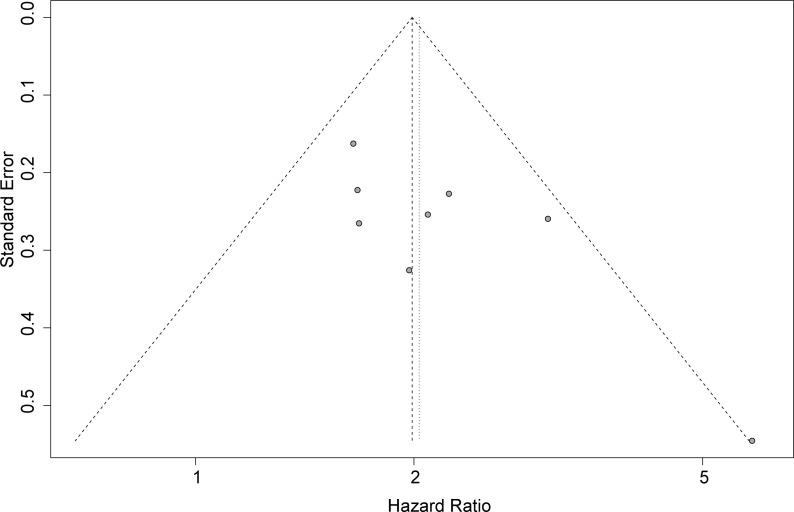
Funnel plot for detecting publication bias.

**Figure 5:**
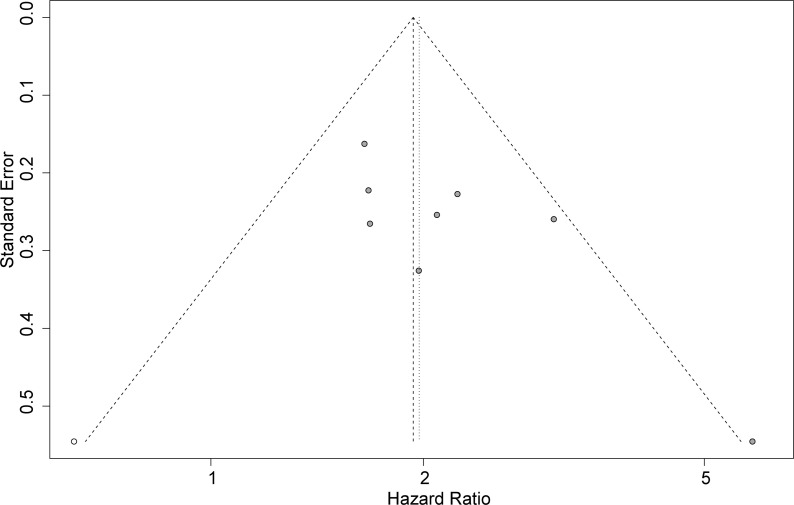
Funnel plot after a trim-and-fill analysis.

Funnel plots, Begg’s test, and Egger’s test were applied to assess the publication bias. The funnel plot for OS was basically symmetrical (**Figure 4**), and the results of Begg’s test showed no significant publication bias (p = 0.0833). However, Egger’s test suggested that there might be publication bias (p = 0.0424). Therefore, we conducted a trim-and-fill analysis to modify our primary results, and the results confirmed that our meta-analysis was robust even after eliminating the impact of publication bias with the funnel plot being basically symmetrical (HR = 1.93, 95% CI: 1.64–2.29, p < 0.0001) (**Figure 5**).

## Discussion

In the present study, we aimed to evaluate the prognostic value of GNRI in lung cancer patients, and the pooled results revealed that the lower GNRI was significantly associated with OS, CSS, and RFS in lung cancer patients. Moreover, these results remained stable even after subgroup analysis and sensitivity analyses for detecting underlying confounders, which suggested that a lower pretreatment GNRI was an independent indicator for worse prognosis in lung cancer patients. To the best of our knowledge, this is the first meta-analysis that comprehensively explored the impact of GNRI on non-small cell lung cancer.

Malnutrition is important to the immune system and related to inflammation and cachexia, which can even lead to the mortality of many diseases including tumors (24–26). Hence, several biomarkers were designed to identify the malnutrition of patients, including PNI (27) and CONUT (28); nevertheless, these indices lacked worth of use in elderly patients because of limitations in usual weight estimation. Therefore, GNRI was made and served as an age-specific index to evaluate the nutritional status of elderly patients, which consists of two parameters including serum albumin and actual weight-to-ideal weight ratio. Surprisingly, current studies proposed that GNRI might have better prognostic value rather than nutrition assessment in many diseases, including heart failure (29, 30), hemodialysis (31), and especially malignancies (32–34). Furthermore, GNRI is also capable of predicting postoperative complications in patients with several types of cancers (35, 36). Altogether, GNRI can be a promising predictor for adverse outcome in tumor patients, and thus we focused on its impact on lung cancer. Two previous meta-analyses also discussed the association between GNRI and human cancer (10, 11). The study by Lv et al. (10) revealed that a lower pretreatment GNRI was associated with poorer OS, progression-free survival (PFS), RFS, and CSS in cancer patients. However, the heterogeneity among studies was extremely high, and the number of articles for each kind of tumor was so few that there was only one study about lung cancer. The study by Xie et al. (11) evaluated the effect of GNRI on outcomes of patients with gastrointestinal malignancy, and their results showed that a lower GNRI was associated with worse outcomes of OS and RFS instead of CSS. Certainly, these pieces of evidence were consistent with our meta-analysis. However, the underlying mechanism still needs to be elucidated, and we speculate about the reasons according to the two compositions of GNRI because previous studies demonstrated that lower serum albumin level and low BMI can affect the prognosis of lung cancer patients (37, 38). Surely, there are some limitations of our study. The most obvious one is that the number of the studies we included was few (n = 8), and especially for CSS and RFS (n = 2). However, our including and excluding criteria were strict and the heterogeneity among included studies was high, which did not attenuate the reliability of our meta-analysis. The second one is that we did not retrieve any study carried out beyond Asia, thus the results may not be practical for patients of other ethnicities. Third, the included studies were almost retrospective studies, and this may contribute to the heterogeneity of our study.

Taken together, we found that a lower GNRI was an independent indicator of poorer prognosis in lung cancer patients. However, these findings must be applied seriously in clinical practice, and more prospective cohort studies are needed to confirm our results.

## Data Availability Statement

The original contributions presented in the study are included in the article/**Supplementary Material**. Further inquiries can be directed to the corresponding author.

## Author Contributions

WL and DL contributed to conceptualization and supervision. HW formulated the study question and objectives and planned the search strategy. HW and RY carried out the primary search and data extraction. HW performed the statistical analysis and data interpretation and wrote the primary article. CL, JJ, DL, and WL reviewed the article. All authors read and approved the final article. All authors contributed to the article and approved the submitted version.

## Funding

This work was supported by the National Natural Science Foundation of China (grant numbers 91859203 and 81871890) and Major Science and Technology Innovation Project of Chengdu City (grant number 2020-YF08-00080-GX).

## Conflict of Interest

The authors declare that the research was conducted in the absence of any commercial or financial relationships that could be construed as a potential conflict of interest.

## Publisher’s Note

All claims expressed in this article are solely those of the authors and do not necessarily represent those of their affiliated organizations, or those of the publisher, the editors and the reviewers. Any product that may be evaluated in this article, or claim that may be made by its manufacturer, is not guaranteed or endorsed by the publisher.
