# Supplementary material for: Prognostic Value of the Geriatric Nutritional Risk Index in Non-Small Cell Lung Cancer Patients: A Systematic Review and Meta-Analysis
Source: Front Oncol. 2022 Jan 18;11:794862. doi: 10.3389/fonc.2021.794862 (PMC8804216; doi:10.3389/fonc.2021.794862)
Supplement: Supplementary file 2 [file DataSheet_2.docx]

**Supplementary File 2**

**Search strategy for meta-analysis (PubMed via NLM)**

Search terms: geriatric nutritional risk index and lung cancer patients

Population: lung cancer patients

#1: lung neoplasms[MeSH] OR Pulmonary Neoplasms OR Neoplasms, Lung OR Lung Neoplasm OR Neoplasm, Lung OR Neoplasms, Pulmonary OR Neoplasm, Pulmonary OR Pulmonary Neoplasm OR Lung Cancer OR Cancer, Lung OR Cancers, Lung OR Lung Cancers OR Pulmonary Cancer OR Cancer, Pulmonary OR Cancers, Pulmonary OR Pulmonary Cancers OR Cancer of the Lung OR Cancer of Lung OR Adenocarcinoma of Lung[MeSH]

Exposure: geriatric nutritional risk index

#2: geriatric nutritional risk index OR GNRI

Combined sets: #1 AND #2
